# The Cardiometabolic Burden of Self-Perceived Obesity: A Multilevel Analysis of a Nationally Representative Sample of Korean Adults

**DOI:** 10.1038/s41598-018-26192-z

**Published:** 2018-05-21

**Authors:** Yongjoo Kim, S. Bryn Austin, S V Subramanian, Ichiro Kawachi

**Affiliations:** 1 Department of Social and Behavioral Sciences, Harvard T.H. Chan School of Public Health, Boston, Massachusetts, USA; 2 Division of Adolescent and Young Adult Medicine, Boston Children’s Hospital, Boston, Massachusetts, USA

## Abstract

Emerging evidence has shown that self-perception of overweight/obese status is associated with unfavorable cardiometabolic outcomes, above and beyond actual body weight. Given the lack of research among Asian populations, we examined the association between weight perception and metabolic syndrome (MetS) and cardiometabolic risks among Koreans. Data from the 2010–2015 Korea National Health and Nutrition Examination Survey, including women (N = 12,181) and men (N = 9,448) aged 19–65 years, were analyzed. Weight status perception was measured by participants’ self-evaluation of their body size (“very/slightly obese,” “normal,” and “very/slightly thin”). Overall, 23.2% of women and 28.7% of men had MetS. Our cross-sectional multilevel logistic analyses showed a significant positive association between self-perceived obesity (vs. perceived normal weight) and MetS, independent of BMI and sociodemographic/behavioral/medical conditions, with a stronger association detected among men (OR = 1.38, p < 0.05) than women (OR = 1.22, p < 0.05), confirmed by a statistically significant interaction. Additionally, perceived obesity was associated with high blood pressure (OR = 1.27, p < 0.05) and high triglycerides (OR = 1.38, p < 0.05) among men and low high-density lipoprotein cholesterol (OR = 1.15, p < 0.05) among women. While further prospective research is needed, our findings suggest that perception of being obese may be an unfavorable indicator of cardiometabolic health among Koreans regardless of actual body weight.

## Introduction

Self-perception of body weight has been linked with psychological, behavioral, and biological outcomes above and beyond actual body weight. Among individuals with normal weight, accurate weight perception (i.e., perception of being normal weight) has been consistently linked with more favorable health outcomes compared to weight misperception^1,2^. However, among individuals with overweight/obesity, the findings have been inconsistent. Some studies have reported that individuals with overweight/obesity who misperceived their weight status as being normal were less likely to have intentions and make attempts to control their weight than their counterparts who accurately perceived their weight status, suggesting that accurate weight perception may be an essential component of successful obesity prevention and weight management^3–5^. This perspective has been either implicitly or explicitly adopted in awareness-raising strategies in public health and clinical practices such as school-based obesity screening with notification of the results through body-mass index (BMI) report cards and weight-loss counseling seeking to motivate weight control by accurately informing patients of their overweight/obese status^6–8^.

Recent research, however, has revealed contradictory findings, whereby weight misperception (vs. accurate perception) among individuals with overweight/obesity led to more favorable outcomes in weight gain^9–11^, depressive symptoms^10,12^, and cardiometabolic functioning^10,13^. Furthermore, in a recent systematic review, Haynes and colleagues (2018) concluded that perception of obesity (vs. perception of normal weight) is associated with more unfavorable weight-related outcomes such as disordered eating, unhealthy weight control practice, and weight gain, regardless of the accuracy of the perception^14^. These studies suggested that individuals who identify their weight status as being overweight/obese may be more vulnerable to the negative psychological processes related with weight-based stigma and discrimination^8–15^, which in turn can lead to chronic stress reactions, maladaptive coping behaviors, and biological alterations^16–18^. Other studies also suggested that perception of being overweight/obese may be associated with greater use of disordered weight control behaviors (DWCB) including fasting, self-induced vomiting, and taking laxatives/diuretics^14^. From this viewpoint, investigators have proposed that awareness-oriented approaches in obesity prevention and weight management could stigmatize individuals due to their body weight and impose psychological and metabolic burdens^9–14^.

In South Korea, the population metabolic health indicators have worsened over the past decade. For instance, an increasing trend in the prevalence of obesity was reported among Korean adults (i.e., from 26.9% in 1998 to 32.0% in 2011)^19^. Moreover, the prevalence of metabolic syndrome (MetS), the clustering of cardiometabolic risk factors (CMRs) such as insulin resistance, central obesity, hypertension, and dyslipidemia, sharply increased from 24.9% in 1998 to 31.3% in 2007 among adults^20^. However, gaps exist in the current understanding of the association between weight perception and cardiometabolic risks among Asian populations. For instance, despite the emerging evidence showing the harmful influences of accurate weight perception on cardiometabolic outcomes among individuals with obesity in Western countries^9–11,13^, previous Korean studies generally framed weight misperception as a cognitive bias that should be corrected regardless of weight status^21–23^.

Thus, in the present study, we investigated the association of weight perception with MetS and CMRs among Korean adults. Based on prior literature, we hypothesized that perception of being obese (vs. perception of being normal weight status) would be positively associated with MetS and CMRs among Koreans. We also examined whether the association differed across gender and weight status. Based on previous evidence showing greater weight-based discrimination directed toward women than men^24,25^, we expected that the association between weight perception and MetS and CMRs would be stronger among women than men.

## Results

### Descriptive Statistics

As shown in Table 1, the mean age of the subjects was 44.7 (SD = 12.5) years (45.0 years in women and 44.3 years in men), and the mean BMI was 23.9 (SD = 3.3) kg/m^2^ (24.5 kg/m^2^ in women and 23.5 kg/m^2^ in men). The prevalences of MetS and CMRs were 25.6% for MetS (23.2% in women and 28.7% in men), 27.9% for high fasting plasma glucose (22.2% in women and 35.3% in men), 32.4% for high blood pressure (26.1% in women and 40.4% in men), 35.4% for low high-density lipoprotein cholesterol (41.8% in women and 27.0% in men), 28.9% for high triglyceride (19.8% in women and 40.6% in men), and 33.0% for high waist circumference (37.7% in women and 27.0% in men).

**Table 1:** Descriptive Characteristics of the Study Sample: Korea National Health and Nutrition Examination Survey 2010–2015.

Variables	N (%)		
	All (N = 21,629)	Women (N = 12,181)	Men (N = 9,448)
MetS	5,534 (25.6)	2,826 (23.2)	2,708 (28.7)
High FPG	6,034 (27.9)	2,699 (22.2)	3,335 (35.3)
High BP	7,001 (32.4)	3,181 (26.1)	3,820 (40.4)
Low HDL-C	7,647 (35.4)	5,097 (41.8)	2,550 (27.0)
High TG	6,249 (28.9)	2,416 (19.8)	3,833 (40.6)
Perceived obesity	10,352 (47.9)	6,310 (51.8)	4,042 (42.8)
Perceived normal weight	8,697 (40.2)	5,017 (41.2)	3,680 (39.0)
Perceived underweight	2,580 (11.9)	854 (7.0)	1,726 (18.3)
DWCB	1,875 (8.7)	1,356 (11.1)	519 (5.5)
Menopause		4,846 (39.8)	
Never married	3,826 (17.7)	1,813 (14.9)	2,013 (21.3)
Married	16,255 (75.2)	9,190 (75.5)	7,065 (74.8)
Separated/divorced/widowed	1,548 (7.2)	1,178 (9.7)	370 (3.9)
Education: < = Middle school graduate	5,098 (23.6)	3,379 (27.7)	1,719 (18.2)
High school graduate	8,474 (39.2)	4,741 (38.9)	3,733 (39.5)
> = College graudate	8,057 (37.3)	4,061 (33.3)	3,996 (42.3)
Household income: lowest	2,118 (9.8)	1,319 (10.8)	799 (8.5)
mid-low	5,493 (25.4)	3,176 (26.1)	2,317 (24.5)
mid-high	6,816 (31.5)	3,761 (30.9)	3,055 (32.3)
highest	7,202 (33.3)	3,925 (32.2)	3,277 (34.7)
Urbanicity: Rural	3,604 (16.7)	1,934 (15.9)	1,670 (17.7)
Urban	18,025 (83.3)	10,247 (84.1)	7,778 (82.3)
Depression	879 (4.1)	699 (5.8)	180 (1.9)
Severe chronic conditions	1,184 (5.5)	722 (5.9)	462 (4.9)
Current smoker	4,733 (21.9)	627 (5.2)	4,106 (43.5)
Former smoker	3,423 (15.8)	432 (3.6)	2,991 (31.7)
High-risk drinking	2,769 (12.8)	622 (5.1)	2,147 (22.7)
Physical activity (> = 3days/week)	3,793 (17.5)	1,959 (16.1)	1,834 (19.4)
Survey year: 2010	4,002 (18.5)	2,219 (18.2)	1,783 (18.9)
2011	3,911 (18.1)	2,194 (18.0)	1,717 (18.2)
2012	3,610 (16.7)	2,068 (17.0)	1,542 (16.3)
2013	3,647 (16.9)	2,034 (16.7)	1,613 (17.1)
2014	3,173 (14.7)	1,830 (15.0)	1,343 (14.2)
2015	3,286 (15.2)	1,836 (15.1)	1,450 (15.4)
	Mean (SD)		
Age (years)	44.7 (12.5)	45.0 (12.4)	44.3 (12.6)
BMI (kg/m^2^)	23.9 (3.3)	23.5 (3.3)	24.5 (3.1)

^a^Abbreviations: BMI (body-mass index), BP (blood pressure), CMR (cardiometabolic risks), DWCB (disordered weight control behavior), FPG (fasting plasma glucose), HDL-C (high-density lipoprotein cholesterol), MetS (metabolic syndrome), TG (high triglycerides).

^b^“N” represents raw frequency and “%” indicates column percentage.

### Multilevel Analyses

Our multivariable multilevel models showed that weight perception was significantly associated with MetS and CMRs, but differentially across gender (p-values for interaction tests <0.05, Supplementary Table 1.) Counter to our expectation, generally stronger associations were found among men than women for most of the outcomes, except for low HDL-C. As shown in Tables 2 and 3, perception of being obese (vs. perception of being normal weight) was significantly positively associated with MetS among both women (OR = 1.18, 95% CI: 1.04, 1.35, p < 0.05) and men (OR = 1.42, 95% CI: 1.24, 1.63, p < 0.05), after adjusting for BMI, sociodemographic factors, and survey year (Model 1). The direction and statistical significance of these relationships remained consistent after further adjusting for comorbidity (Model 2) and behavioral factors (smoking/drinking/physical activity, Model 3), and DWCB (Model 4).

**Table 2:** Association of Weight Status Perception with Metabolic Syndrome and Cardiometabolic Risks Among Women (N = 12,181).

	OR (95% CI)			
	Model 1	Model 2	Model 3	Model 4
MetS				
Perceived obesity (vs. normal weight)	**1**.**18 (1**.**04**, **1**.**35)**	**1**.**19 (1**.**01**, **1**.**42)**	**1**.**18 (1**.**01**, **1**.**37)**	**1**.**22 (1**.**04**, **1**.**43)**
Perceived underweight	1.07 (0.79, 1.42)	1.08 (0.78, 1.46)	1.07 (0.80, 1.43)	1.07 (0.77, 1.47)
DWCB				**0**.**66 (0**.**52**, **0**.**83)**
High BP				
Perceived obesity (vs. normal weight)	0.96 (0.85, 1.09)	0.96 (0.84, 1.09)	0.94 (0.82, 1.07)	0.95 (0.84, 1.08)
Perceived underweight	1.07 (0.86, 1.30)	1.07 (0.87, 1.29)	1.06 (0.86, 1.29)	1.05 (0.86, 1.30)
DWCB				0.86 (0.72, 1.02)
High FPG				
Perceived obesity (vs. normal weight)	1.07 (0.94, 1.22)	1.07 (0.93, 1.22)	1.06 (0.92, 1.21)	1.06 (0.92, 1.21)
Perceived underweight	**1**.**38 (1**.**12**, **1**.**71)**	**1**.**39 (1**.**12**, **1**.**70)**	**1**.**36 (1**.**10**, **1**.**69)**	**1**.**36 (1**.**10**, **1**.**69)**
DWCB				0.95 (0.80, 1.10)
Low HDL-C				
Perceived obesity (vs. normal weight)	1.11 (0.98, 1.25)	1.10 (0.97, 1.25)	**1**.**14 (1**.**00**, **1**.**30)**	**1**.**15 (1**.**01**, **1**.**33)**
Perceived underweight	1.10 (0.88, 1.37)	1.11 (0.90, 1.36)	1.10 (0.90, 1.37)	1.13 (0.94, 1.36)
DWCB				**0**.**81 (0**.**69**, **0**.**94)**
High TG				
Perceived obesity (vs. normal weight)	1.03 (0.90, 1.17)	1.01 (0.88, 1.16)	1.00 (0.86, 1.18)	0.99 (0.86, 1.14)
Perceived underweight	0.93 (0.71, 1.22)	0.93 (0.72, 1.18)	0.92 (0.70, 1.21)	0.92 (0.71, 1.18)
DWCB				**0**.**82 (0**.**69**, **0**.**98)**

^a^Abbreviations: BMI (body-mass index), BP (blood pressure), DWCB (disordered weight control behavior), FPG (fasting plasma glucose), HDL-C (high-density lipoprotein cholesterol), MetS (metabolic syndrome), TG (high triglycerides).

^b^All models were based on four-level multilevel random intercepts logistic models, in which individuals at level 1 were nested within households at level 2, nested within neighborhoods at level 3, and nested within geographic areas at level 4.

^c^Model 1 adjusted for age (year), BMI (kg/m^2^), menopause, education, income, marital status, region, and survey year.

^d^Model 2 further adjusted for depression and severe chronic condition.

^e^Model 3 further adjusted for smoking, high-risk drinking, and physical activity.

^f^Model 4 further adjusted for DWCB.

^g^Boldface indicates statistical significance (p < 0.05).

**Table 3:** Association of Weight Status Perception with Metabolic Syndrome and Cardiometabolic Risks Among Men (N = 9,448).

	OR (95% CI)			
	Model 1	Model 2	Model 3	Model 4
MetS				
Perceived obesity (vs. normal weight)	**1**.**42 (1**.**24**, **1**.**63)**	**1**.**54 (1**.**28**, **1**.**92)**	**1**.**37 (1**.**20**, **1**.**56)**	**1**.**38 (1**.**19**, **1**.**59)**
Perceived underweight	1.17 (0.94, 1.47)	1.19 (0.92, 1.56)	1.15 (0.91, 1.45)	1.14 (0.90, 1.45)
DWCB				0.93 (0.73, 1.17)
High BP				
Perceived obesity (vs. normal weight)	**1**.**27 (1**.**12**, **1**.**44)**	**1**.**29 (1**.**13**, **1**.**45)**	**1**.**25 (1**.**10**, **1**.**42)**	**1**.**27 (1**.**13**, **1**.**44)**
Perceived underweight	1.00 (0.85, 1.19)	1.00 (0.86, 1.17)	1.01 (0.86, 1.20)	1.02 (0.86, 1.20)
DWCB				0.87 (0.70, 1.05)
High FPG				
Perceived obesity (vs. normal weight)	1.07 (0.93, 1.22)	1.05 (0.92, 1.20)	1.03 (0.92, 1.17)	1.05 (0.92, 1.20)
Perceived underweight	0.93 (0.78, 1.10)	0.92 (0.77, 1.09)	0.93 (0.78, 1.11)	0.94 (0.80, 1.10)
DWCB				**0**.**76 (0**.**61**, **0**.**94)**
Low HDL-C				
Perceived obesity (vs. normal weight)	1.06 (0.92, 1.21)	1.06 (0.92, 1.22)	1.11 (0.94, 1.29)	1.08 (0.94, 1.25)
Perceived underweight	0.97 (0.80, 1.17)	0.97 (0.80, 1.19)	0.94 (0.77, 1.15)	0.95 (0.77, 1.15)
DWCB				0.91 (0.73, 1.12)
High TG				
Perceived obesity (vs. normal weight)	**1**.**42 (1**.**24**, **1**.**63)**	**1**.**54 (1**.**28**, **1**.**92)**	**1**.**37 (1**.**20**, **1**.**56)**	1.38 (1.19, 1.59)
Perceived underweight	1.17 (0.94, 1.47)	1.19 (0.92, 1.56)	1.15 (0.91, 1.45)	1.14 (0.90, 1.45)
DWCB				0.93 (0.73, 1.17)

^a^Abbreviations: BMI (body-mass index), BP (blood pressure), DWCB (disordered weight control behavior), FPG (fasting plasma glucose), HDL-C (high-density lipoprotein cholesterol), MetS (metabolic syndrome), TG (high triglycerides).

^b^All models were based on four-level multilevel random intercepts logistic models, in which individuals at level 1 were nested within households at level 2, nested within neighborhoods at level 3, and nested within geographic areas at level 4.

^c^Model 1 adjusted for age (year), BMI (kg/m^2^), education, income, marital status, region, and survey year.

^d^Model 2 further adjusted for depression and severe chronic condition.

^e^Model 3 further adjusted for smoking, high-risk drinking, and physical activity.

^f^Model 4 further adjusted for DWCB.

^g^Boldface indicates statistical significance (p < 0.05).

In terms of the association with CMRs, we found that perception of being obese (vs. perception of being normal weight) was significantly associated with high BP (OR = 1.27, 95% CI: 1.12, 1.44, p < 0.05) and high TG (OR = 1.42, 95% CI: 1.24, 1.63, p < 0.05) among men, after adjusting for BMI, sociodemographic factors, and survey year in Model 1. The direction and significance of these associations were consistent after further adjusting for other covariates across different models. However, there was no such relationship for high FPG and low HDL-C among men.

Conversely, among women, perception of being obese (vs. perception of being normal weight) was marginally, but not statistically, significantly associated with low HDL-C (OR = 1.11, 95% CI: 0.98, 1.25, p < 0.10), after adjusting for BMI, sociodemographic factors, and survey year in Model 1. The size of this association slightly increased after further adjusting for behavioral factors such as smoking, drinking, physical activity, and DWCB (OR = 1.15, 95% CI: 1.01, 1.33, p < 0.05). However, we failed to find a significant relationship for other CMRs among women. Notably, perception of being obese (vs. perception of being normal weight) was associated with high FPG (OR = 1.38, 95% CI: 1.12, 1.71, p < 0.05) among women.

Our analysis also showed that DWCB was generally inversely associated with MetS and CMRs, but there were some differences between women and men particularly for MetS and high TG (interaction p-values < 0.05, Supplementary Table 1). For instance, after adjusting for all other covariates, DWCB was inversely associated with MetS (OR = 0.66, 95% CI: 0.52, 0.83, p < 0.05) and high TG (OR = 0.82, 95% CI: 0.69, 0.98, p < 0.05) among women, (Table 2) but not among men (OR for MetS = 0.93, 95% CI: 0.73, 1.17, p > 0.05; OR for high TG = 0.93, 95% CI: 0.73, 1.17, p > 0.05, Table 3).

Tables 4 and 5 showed the results of our sensitivity analyses only among a subset of participants with concordant BMI and waist circumference. In general, the findings between our primary and sensitivity analyses were largely consistent. For instance, perception of being obese (vs. perception of being normal weight) was significantly associated with MetS among both women (OR = 1.27, 95% CI: 1.04, 1.52, p < 0.05) and men (OR = 1.64, 95% CI: 1.39, 1.93, p < 0.05), after adjusting for BMI, sociodemographic factors, and survey year. In terms of CMRs, perception of being obese (vs. perception of being normal weight) was significantly associated with high BP (OR = 1.24, 95% CI: 1.07, 1.44, p < 0.05) and high TG (OR = 1.22, 95% CI: 1.06, 1.40, p < 0.05) among men. Although we failed to find evidence of significant association between perception of being obese and low HDL-C among women in some models (i.e., OR = 1.14, 95% CI: 0.97, 1.36, p > 0.05 in Model 1), the direction of the associations were consistent with our primary analysis across all models.

**Table 4:** Sensitivity Analysis of the Association Between Weight Perception and Metabolic Syndrome and Cardiometabolic Risks Among Women with Concordant BMI and Waist Circumference Categories (N = 10,174).

	OR (95% CI)			
	Model 1	Model 2	Model 3	Model 4
MetS				
Perceived obesity (vs. normal weight)	**1**.**27 (1**.**04**, **1**.**52)**	**1**.**24 (1**.**02**, **1**.**51)**	**1**.**26 (1**.**03**, **1**.**56)**	**1**.**27 (1**.**07**, **1**.**54)**
Perceived underweight	**1**.**51 (1**.**07**, **2**.**14)**	**1**.**52 (1**.**08**, **2**.**13)**	**1**.**54 (1**.**03**, **2**.**29)**	**1**.**51 (1**.**07**, **2**.**14)**
DWCB				**0**.**74 (0**.**60**, **0**.**91)**
High BP				
Perceived obesity (vs. normal weight)	0.87 (0.75, 1.02)	0.86 (0.72, 1.03)	0.88 (0.74, 1.03)	0.88 (0.74, 1.03)
Perceived underweight	1.10 (0.87, 1.38)	1.10 (0.87, 1.42)	1.08 (0.85, 1.36)	1.08 (0.86, 1.34)
DWCB				0.84 (0.69, 1.03)
High FPG				
Perceived obesity (vs. normal weight)	1.11 (0.95, 1.28)	1.12 (0.96, 1.30)	1.08 (0.93, 1.27)	1.12 (0.95, 1.30)
Perceived underweight	**1**.**43 (1**.**14**, **1**.**80)**	**1**.**42 (1**.**11**, **1**.**77)**	**1**.**43 (1**.**12**, **1**.**79)**	**1**.**42 (1**.**12**, **1**.**80)**
DWCB				0.98 (0.81, 1.17)
Low HDL-C				
Perceived obesity (vs. normal weight)	1.14 (0.97, 1.36)	1.14 (0.98, 1.34)	**1**.**16 (1**.**01**, **1**.**43)**	1.16 (1.00, 1.34)
Perceived underweight	1.17 (0.93, 1.45)	1.19 (0.94, 1.53)	1.17 (0.92, 1.46)	1.14 (0.92, 1.44)
DWCB				**0**.**83 (0**.**69**, **0**.**98)**
High TG				
Perceived obesity (vs. normal weight)	1.16 (0.98, 1.39)	1.15 (0.93, 1.42)	1.11 (0.94, 1.30)	1.13 (0.93, 1.37)
Perceived underweight	1.05 (0.78, 1.44)	1.03 (0.74, 1.43)	1.02 (0.76, 1.36)	1.04 (0.74, 1.44)
DWCB				0.84 (0.67, 1.06)

^a^Abbreviations: BMI (body-mass index), BP (blood pressure), DWCB (disordered weight control behavior), FPG (fasting plasma glucose), HDL-C (high-density lipoprotein cholesterol), MetS (metabolic syndrome), TG (high triglycerides).

^b^All models were based on four-level multilevel random intercepts logistic models, in which individuals at level 1 were nested within households at level 2, nested within neighborhoods at level 3, and nested within geographic areas at level 4.

^c^Model 1 adjusted for age (year), BMI (kg/m^2^), menopause, education, income, marital status, region, and survey year.

^d^Model 2 further adjusted for depression and severe chronic condition

^e^Model 3 further adjusted for smoking, high-risk drinking, and physical activity

^f^Model 4 further adjusted for DWCB

^g^Boldface indicates statistical significance (p < 0.05).

**Table 5:** Sensitivity Analysis of the Association Between Weight Perception and Metabolic Syndrome and Cardiometabolic Risks Only Among Men (N = 7,631) with Concordant BMI and Waist Circumference Categories.

	OR (95% CI)			
	Model 1	Model 2	Model 3	Model 4
MetS				
Perceived obesity (vs. normal weight)	**1**.**64 (1**.**39**, **1**.**93)**	**1**.**66 (1**.**40**, **1**.**96)**	**1**.**58 (1**.**34**, **1**.**88)**	**1**.**73 (1**.**42**, **2**.**19)**
Perceived underweight	**1**.**42 (1**.**13**, **1**.**79)**	**1**.**47 (1**.**15**, **1**.**86)**	**1**.**44 (1**.**13**, **1**.**86)**	**1**.**53 (1**.**17**, **2**.**02)**
DWCB				0.90 (0.66, 1.22)
High BP				
Perceived obesity (vs. normal weight)	**1**.**24 (1**.**07**, **1**.**44)**	**1**.**23 (1**.**06**, **1**.**43)**	**1**.**18 (1**.**01**, **1**.**39)**	**1**.**19 (1**.**02**, **1**.**38)**
Perceived underweight	1.02 (0.85, 1.20)	1.03 (0.87, 1.22)	1.04 (0.87, 1.23)	1.03 (0.88, 1.22)
DWCB				0.98 (0.86, 1.12)
High FPG				
Perceived obesity (vs. normal weight)	1.03 (0.88, 1.23)	1.03 (0.88, 1.20)	1.00 (0.85, 1.16)	1.02 (0.86, 1.22)
Perceived underweight	0.98 (0.82, 1.16)	1.00 (0.85, 1.19)	0.98 (0.84, 1.17)	0.99 (0.83, 1.18)
DWCB				0.81 (0.64, 1.03)
Low HDL-C				
Perceived obesity (vs. normal weight)	1.09 (0.91, 1.27)	1.10 (0.94, 1.28)	1.11 (0.93, 1.30)	1.12 (0.93, 1.32)
Perceived underweight	0.96 (0.79, 1.18)	0.96 (0.78, 1.15)	0.94 (0.78, 1.14)	0.93 (0.77, 1.13)
DWCB				0.95 (0.74, 1.22)
High TG				
Perceived obesity (vs. normal weight)	**1**.**24 (1**.**06**, **1**.**44)**	**1**.**25 (1**.**05**, **1**.**45)**	1.20 (1.00, 1.46)	1.18 (1.00, 1.41)
Perceived underweight	0.93 (0.78, 1.12)	0.94 (0.79, 1.11)	0.89 (0.73, 1.10)	0.96 (0.80, 1.12)
DWCB				1.02 (0.80, 1.33)

^a^Abbreviations: BMI (body-mass index), BP (blood pressure), DWCB (disordered weight control behavior), FPG (fasting plasma glucose), HDL-C (high-density lipoprotein cholesterol), MetS (metabolic syndrome), TG (high triglycerides).

^b^All models were based on four-level multilevel random intercepts logistic models, in which individuals at level 1 were nested within households at level 2, nested within neighborhoods at level 3, and nested within geographic areas at level 4.

^c^Model 1 adjusted for age (year), BMI (kg/m^2^), education, income, marital status, region, and survey year.

^d^Model 2 further adjusted for depression and severe chronic condition.

^e^Model 3 further adjusted for smoking, high-risk drinking, and physical activity.

^f^Model 4 further adjusted for DWCB.

^g^Boldface indicates statistical significance (p < 0.05).

It is notable that, in our sensitivity analysis, perception of being underweight (vs. perception of being normal weight) was significantly positively associated with MetS in both women (OR = 1.51, 95% CI: 1.07, 2.14, p < 0.05) and men (OR = 1.42, 95% CI: 1.13, 1.79, p < 0.05) and high FPG in women (OR = 1.43, 95% CI: 1.14, 1.80, p < 0.05).

Our analysis of interactions between weight perception and weight status among women and men, separately, showed that there was no evidence of significant interaction except for high blood pressure (interaction p-value <0.05) among men (Supplementary Tables 2 and 3).

## Discussion

Given the lack of understanding regarding the linkage between weight perception and cardiometabolic health in Asian countries, the present study examined whether weight perception was cross-sectionally associated with MetS and CMRs among a nationally representative sample of Korean adults. We found that individuals who identified their weight status as being slightly/very obese (vs. normal weight) were 18–54% more likely to meet the criteria of MetS, independent of their actual body weight and other sociodemographic/medical/behavioral factors. Counter to our expectation, the size of the association was greater among men than women. Moreover, the association with specific CMR differed across gender. Among men, those who self-classified their weight as being obese (vs. normal) were 25–29% more likely to have high BP and 37–54% more likely to have high TG. Among women, individuals who described their weight as being obese (vs. normal) were 10–15% more likely to have low HDL-C. These associations did not differ across weight status for all outcomes, except for high BP in men. To our knowledge, this study is the first to document such relationships among Koreans.

Our results are consistent with the findings from recent studies showing that perception of normal weight (i.e., weight underperception) was associated with lower systolic blood pressure^13^ and more favorable physiological functioning (i.e., a composite measure of inflammatory, cardiovascular, and metabolic biomarkers)^10^ among US youth with overweight/obesity. While these studies found no difference in the association across gender^10,13^, we found a stronger association between weight perception and MetS among men than women. This is contrary to our initial hypothesis that perceived normal weight (vs. perceived obesity) would be more protective among women than men given that fat bias has been found to be more strongly directed toward women than men^24,25^.

Moreover, when it comes to CMR, we found different patterns across gender, whereby significant associations were found for high BP and high TG among men, whereas a significant association was detected for low HDL-C among women. Given the prior evidence connecting perceived obesity with MetS among individuals with normal weight^26^, our study replicates and extends the previous research by documenting that dyslipidemia (i.e., low HDL-C in women and high TG in men) and high BP could drive the overall association between weight perception and MetS.

In a broader sense, our findings are also in line with previous evidence showing a protective association of perception of normal weight (i.e., weight underperception vs. accurate perception) with weight change^9–11^ and depressive symptoms^12^ among adolescents and young adults with overweight/obesity. Similarly, our findings also align with a previous prospective study documenting a positive association between weight overperception (vs. accurate perception) and risk of weight gain and obesity among adolescents with normal weight^2^.

To understand how self-assessment of weight status can be associated with cardiometabolic and psychological health outcomes, recent studies have focused on emotional responses related with perception of body size^6–15,27,28^. That is, studies have suggested that, considering negative societal values and images attached to obesity, self-evaluation of being obese could be associated with weight bias internalization, low self-esteem, and body dissatisfaction^8–15,27,28^. An accumulated body of research has shown that weight stigma is prevalent in a number of populations across diverse social groups^16,29–32^, and weight discrimination can induce sustained psychological distress and maladaptive coping behaviors, which can lead to biological alterations such as prolonged hyperactivation of the hypothalamic-pituitary-adrenal axis and cortisol secretion, increased weight gain, and dysregulation of inflammatory and cardiometabolic processes^10,17,18,33^.

Additionally, recent studies have found that being identified as overweight/obese by others (i.e., weight labeling) and by BMI report cards through BMI screening is associated with increased weight gain among US female youth^34,35^. By inter-relating weight stigma, identification of overweight/obese status by others or BMI report cards, and self-perception of being obese, recent studies have suggested that individuals who accurately perceive their overweight/obese status might experience negative psychological processes related with weight stigma and body dissatisfaction, which can result in weight gain and cardiometabolic dysregulations^8–10,13,27^.

Recent studies have shown that weight bias may be prevalent in South Korea^24^. While a plump body was favored in the traditional Korean society, the rapid economic and sociocultural changes reshaped the normative body image, bringing out the widespread thin-ideal in Koreans^24,36–38^. This was supported by recent studies showing that Koreans hold the strongest preference for individuals with thin body (vs. individuals with obesity) among all participants globally from the seventy-two countries involved in the Project Implicit^39^. Moreover, the prevalence of disordered weight control behaviors and body dissatisfaction among Korean adolescents and young adults were found to be comparable to that among US adolescents and young adults^40,41^. As proposed by Robinson (2017), it is possible that Koreans with perception of obesity may experience higher levels of psychological distress due to their perception of belonging to a socially stigmatized group, which can be detrimental to psychological and cardiometabolic well-being^28^.

Additionally, we also found that women who identified themselves as being slightly/very thin (vs. normal weight) were 36–38% more likely to have high FPG. Previous prospective and cross-sectional studies documented that perceived underweight (vs. normal weight) was associated with increased risk of depression among adolescents^1,12,42^ and adults^43^. This study is the first to report an association with cardiometabolic outcomes. It has been suggested that perception of underweight may be related to body image distortion and dissatisfaction, reflecting a desire for masculinity particularly among adolescent and young adult men^42–46^. However, further research is warranted to verify and understand such association with cardiometabolic outcomes in women as well as men.

Contrary to prior evidence showing a positive association between DWCB and weight gain^2,47–49^, we found that DWCB was generally inversely associated with MetS and CMRs among women and men. Most prior studies, though not all, have found that DWCB (i.e., fasting, skipping meals, self-induced vomiting, and using diet pills) was associated with an elevated risk of subsequent weight gain and obesity^47–49^. Regarding the paradoxical associations between dieting and weight gain, prior studies mainly suggested potential explanations such as food preoccupation and over-/binge eating^47–49^, increased psychological distress^50^, and metabolic adaptation^48,51^. Nevertheless, in other studies, caloric restriction, defined as reduced caloric intake without deprivation of necessary nutrients, has been linked with favorable cardiometabolic outcomes^52–54^. Further prospective studies with more detailed information on dietary behaviors are necessary to understand the association in the Korean context.

Our study has several limitations. First, our cross-sectional design is susceptible to reverse causation. It is possible that individuals with MetS were more likely to have psychological distress and depressive symptoms, which could influence self-perception of weight status and weight control behaviors. Although our models adjusted for an extensive range of variables such as BMI (as a quadratic term), medical history (depression and cancer/stroke/heart attack/renal failure/cirrhosis), and behavioral (smoking, alcohol drinking, and exercise) and sociodemographic profiles, we cannot preclude reverse causality. Additionally, misclassification of weight perception pattern is possible, particularly among those with discrepant BMI and WC values. However, our sensitivity analysis using only individuals with concordant BMI and WC values showed largely consistent associations with our primary analyses. Lastly, while prior studies suggested over-/binge eating as potential behavioral pathways linking weight perception and DWCB with weight gain^27,47^, we were not able to obtain information on over-/binge eating.

However, our study also had the following strengths. To the best of our knowledge, this study is the first to document the associations of weight status perception with CMRs in an Asian sample. We used a large nationally representative sample with objectively measured anthropometric and biochemical information. Additionally, we investigated differential associations across gender and weight category, and extended our analysis to examine CMRs as well as MetS. Lastly, given the well-documented clustering of CMRs within households^55,56^ and geographic localities^57^, we performed four-level multilevel models, incorporating all sampling stages (areas-neighborhoods-households-individuals) as units of analysis, thereby adjusting for clustering induced by the hierarchical data structure. To our knowledge, our analysis is the first to use a full four-level multilevel approach with the KNHANES datasets.

The findings of this study suggest that the self-perception of being obese may be associated with unfavorable cardiometabolic health markers, regardless of actual body weight. That is, while accurate weight perception may be a sign of healthier cardiometabolic functioning than weight misperception among individuals with normal weight, the opposite may be true for individuals with obesity. Our findings support the emerging perspective that raises concerns regarding awareness-oriented approaches for weight management and obesity prevention implemented in clinical and public health practices. At the policy level, obesity prevention strategies that focus on alarming those at elevated risk of obesity such as BMI report cards interventions need to take into account the potential unexpected adverse influences of “accurate” perception among individuals with obesity. In clinical practice, providers may be able to incorporate information on weight perception in risk identification for cardiometabolic dysregulation. As suggested by recent studies, weight loss and obesity prevention approaches may benefit from focusing on lifestyle changes such as healthy eating and physical activity.

## Methods

### Study Population

Data were from the 2010–2015 Korea National Health and Nutrition Examination Survey (KNHANES), conducted by the Korea Centers for Disease Control and Prevention (K-CDC)^58^. With a nationally representative sample, the survey monitors the health, behavioral, and nutritional profiles of Koreans. KNHANES employed a stratified three-stage cluster sampling procedure, including 192 geographic areas (i.e., neighborhood/town/township) as primary sampling units (PSU), 20 households within each PSU as secondary sampling units, and all household members aged 1 year or older as final sampling units^58^.

**Figure 1:**
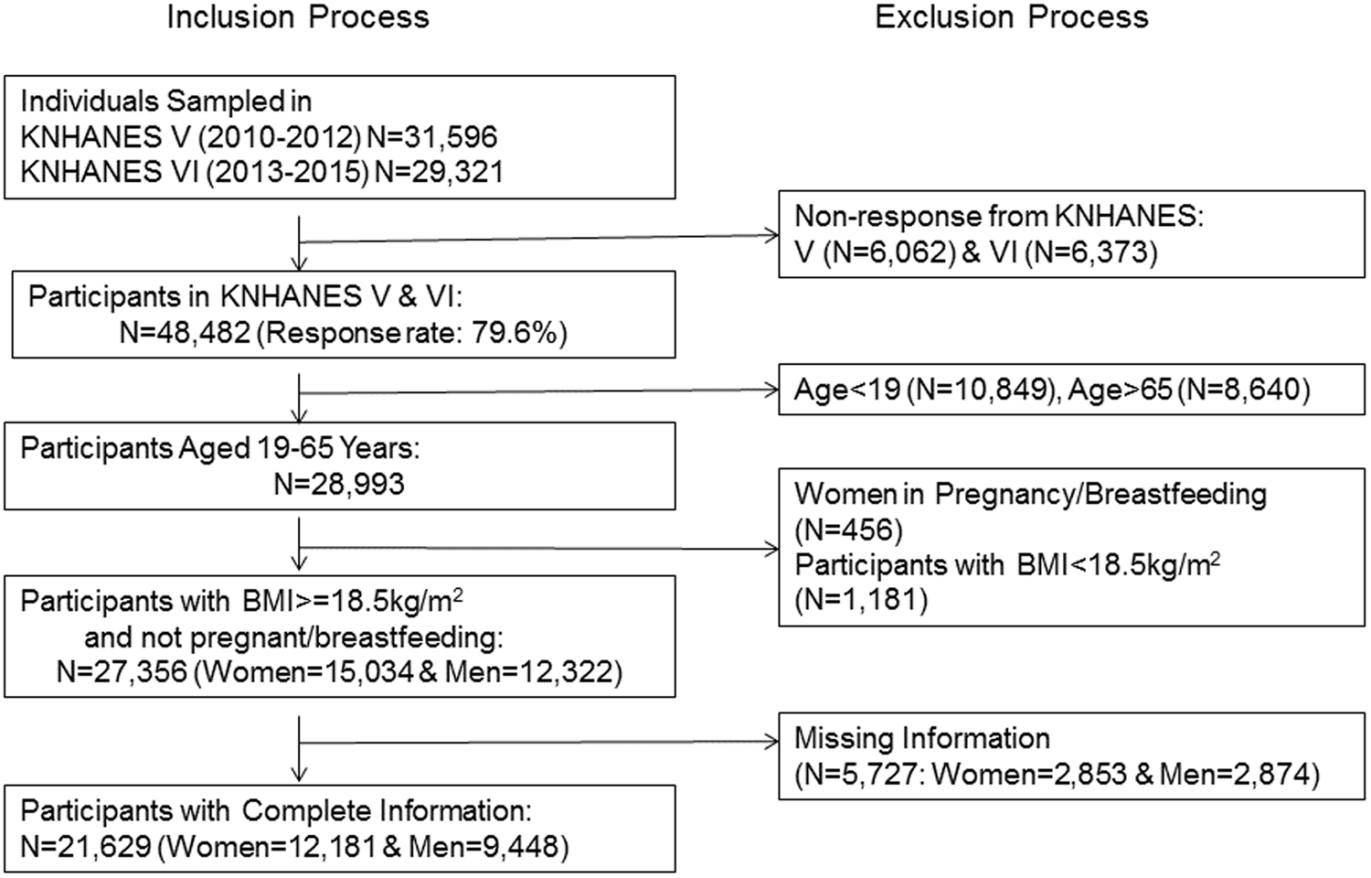
Inclusion and Exclusion Processes for the Study Sample.

Of the 60,917 individuals initially targeted, 48,482 individuals participated in the survey (Fig. 1). Of these, we included adults aged 19–65 years, with BMI > = 18.5 kg/m^2^, not pregnant/breastfeeding, and without any missing information on the variables used in our analyses, yielding an analytic sample of 21,629 individuals (12,181 women and 9,448 men). All participants consented to participate in the survey. Since we used de-identified public-use data, this study received an IRB review exemption by the Office of Human Research Administration at the Harvard T.H. Chan School of Public Health.

### Subject Evaluation and Laboratory Assessment

Anthropometric information was obtained by trained medical staff at mobile examination centers. Height and weight, respectively, were measured with a portable stadiometer (with precision of 1 mm with participants standing barefoot) and electronic scale (with precision of 100 g with participants wearing a light gown). Obesity was defined as a BMI (weight in kilograms divided by height in meters squared) value of 25.0 kg/m^2^ or above, according to the revised criteria for Asian-Pacific populations from the World Health Organization^59,60^. Waist circumference (WC) was measured with a measuring tape (with precision of 1 mm) at the midway between the iliac crest and lower rib margin after normal expiration^58^.

After participants had rested in a sitting position (> = 5 minutes), systolic and diastolic blood pressures (BP) were assessed with a standard mercury sphygmomanometer from the right arm three times each for systolic and diastolic BPs, with a 30-second interval between measurements. Our analysis used the mean values of the second and third assessments of BP based on the K-CDC’s guideline and previous literature^61^.

Blood samples were collected from participants’ antecubital vein after fasting (> = 8 hours), centrifuged and refrigerated at the mobile examination center, then transferred in ice boxes to a central laboratory in Seoul within 24 hours. Triglycerides (TG), fasting plasma glucose (FPG), and high-density lipoprotein cholesterol (HDL-C) were assessed with an automated hematology analyzer at the central laboratory^58^.

### Metabolic Syndrome and Cardiometabolic Risk Factors

MetS was defined, according to the revised definition of the National Cholesterol Education Program-Adult Treatment Panel III, as the presence of > = 3 CMRs: (a) high WC (> = 90 cm for men, > = 80 for women)^62^, (b) high FPG (> = 100 mg/dL, or anti-hyperglycemic agent use), (c) high BP (> = 130/85 mmHg, or antihypertensive agent use), (d) low HDL-C (<40 mg/dL for men, <50 mg/dL for women), and (e) high TG (> = 150 mg/dL)^63^. The criteria for each CMR was based on the Joint Scientific Statement by the International Diabetes Federation, National Heart, Lung, and Blood Institute, and American Heart Association^63^.

### Weight Status Perception

Perceived body weight status was measured with the question, “How would you describe your body shape?” The original responses (“very obese,” “slightly obese,” “normal,” “slightly thin,” and “very thin”) were grouped as “very/slightly obese,” “normal,” and “slightly/very thin.”^58^

### Disordered weight control behaviors (DWCB)

Participants were asked whether they had engaged in the following behaviors for weight control over the past year: fasting, skipping meals, following a one-food diet (i.e., eating only one specific food for diet), and using unprescribed diet pills, with a yes/no response option for each item^58^. Based on prior literature, a composite indicator variable for DWCB was generated to represent whether a participant engaged in at least one of the four behaviors over the past year.

### Covariates Assessment

Educational attainment (middle school or less, high school, and college graduate or higher) was measured based on the participants’ highest academic diploma achieved. Quartiles of equivalized total household income (i.e., self-reported total household income divided by the number of household members squared) was used to measure economic status. Marital status was grouped as married, widowed/divorced/separated, and never married^58^. Smoking behavior was categorized as a non-smoker (lifetime smoking less than 5 packs), former smoker (have not smoked within the last 30 days with lifetime smoking of 5 packs or more), and current smoker (smoked within the last 30 days with lifetime smoking of 5 packs or more). A binary variable for high-risk alcohol use was created, indicating heavy drinking (> = 7 glasses for men and > = 5 glasses for women per each instance), which occurred frequently (> = 2 times/week) over the past 12 months. Regular moderate-level physical activity, a binary variable, was defined as engaging in exercise that moderately elevated breathing or heart rate for 10 minutes or more each instance, for 3 days or more every week. Self-reported information on medical history was used to create indicator variables for physician-diagnosed depression and chronic severe condition (any of stroke/cancer/heart attack/renal failure/cirrhosis)^58^.

### Data Availability

All data used in this study is publicly available upon request via https://knhanes.cdc.go.kr/.

### Statistical Analysis

To understand the association between weight perception pattern and MetS, we constructed four-level multilevel logistic models, where individuals (N = 21,623: level 1) were nested within households (N = 13,182: level 2), nested within neighborhoods (N = 1,152: level 3), and nested within geographic areas (N = 16: level 4), thereby adjusting for the potential clustering of MetS within households and neighborhoods induced by the hierarchical data structure. Based on prior evidence of differences in pathophysiology of MetS by gender^64^ and significant interactions between gender and weight perception in our samples (p-values for interaction tests for MetS and CMRs <0.05, Supplementary Table S1), we constructed gender-stratified models.

A set of covariates was sequentially added to the models: age (year, as a linear term), BMI (kg/m^2^, as a quadratic term), menopause (for women), sociodemographic factors such as education, marital status, household income, urbanicity of residential area, and survey year in Model 1; comorbidity such as history of depression and severe chronic condition (cancer/stroke/coronary heart disease) in Model 2; health-related behaviors smoking, drinking, and exercise in Model 3. Based on prior literature showing an association between weight perception and DWCB^15^ and an association between DWCB and weight gain^47–49^, we further included DWCB in Model 4, based on Model 3, to understand whether DWCB explains the potential association between weight perception and MetS.

To address the potential issue of confounding by actual body weight, we included BMI (kg/m^2^) as a mean-centered quadratic term based on the most favorable (smallest) deviance information criteria statistics from a model with a quadratic BMI compared to models with a categorical or linear continuous BMI. To better understand which specific CMR contributed to the potential association between weight perception and MetS, we examined high FPG, high BP, low HDL-C, and high TG separately as an outcome.

Concerning possible misclassification of weight perception among individuals with discordant BMI and waist circumference (WC) categories (e.g., an individual with high WC but still within the “normal” BMI range), we performed sensitivity analysis by restricting the analyses only to individuals with concordant BMI and WC categories (including 10,174 women with either WC > = 80 cm and BMI > = 25 kg/m^2^ or WC < 80 cm and BMI < 25 kg/m^2^; and 7,631 men with either WC > = 90 cm and BMI > = 25 kg/m^2^ or WC < 90 cm and BMI < 25 kg/m^2^).

As a post-hoc analysis, we tested whether the associations were stronger among individuals with obesity (BMI > = 25.0 kg/m^2^) among women and men separately.

All statistical analyses were performed with MLwiN 3.01 (Bristol University). We employed the Markov Chain Monte Carlo method with the Metropolis-Hastings algorithm based on uninformative priors for multilevel analyses^65^.

## Electronic supplementary material

Supplementary Tables
