# Supplementary material for: The Cardiometabolic Burden of Self-Perceived Obesity: A Multilevel Analysis of a Nationally Representative Sample of Korean Adults
Source: Sci Rep. 2018 May 21;8:7901. doi: 10.1038/s41598-018-26192-z (PMC5962568; doi:10.1038/s41598-018-26192-z)
Supplement: Supplementary file 1 — Supplementary Tables [file 41598_2018_26192_MOESM1_ESM.docx]

**TITLE**: The Cardiometabolic Burden of Self-Perceived Obesity: A Multilevel Analysis of a Nationally Representative Sample of Korean Adults

**AUTHORS**: Yongjoo Kim, ScD, MPH,*^1^ S. Bryn Austin, ScD,^1,2^ S.V. Subramanian, PhD,^1^ Ichiro Kawachi, MD, PhD^1^

**AUTHORS’ AFFILIATIONS**:

^1^Department of Social and Behavioral Sciences, Harvard T.H. Chan School of Public Health, Boston, Massachusetts, U.S.A

^2^Division of Adolescent and Young Adult Medicine, Boston Children’s Hospital, Boston, Massachusetts, U.S.A

**CONTACT INFO:**

Corresponding Author: Yongjoo Kim, ScD, MPH

Mailing Address:

677 Huntington Avenue, 7^th^ Floor Kresge Building,

Harvard T.H. Chan School of Public Health, Boston, MA 02115

Email Address: yongjoo.kim@mail.harvard.edu

**Appendix: Supplementary Tables**

**Table S1**. Analysis of Interaction Between Gender and Weight Perception Based on Multilevel Logistic Models Predicting Metabolic Syndrome and Cardiometabolic Risk Among Korean Adults (N=21,629)

|  | OR  (95% CI) | | | | |
| --- | --- | --- | --- | --- | --- |
|  | MetS | high FPG | high BP | low HDL-C | high TG |
| Perceived Obesity | **1.46**  **(1.30, 1.64)** | **1.17**  **(1.04, 1.30)** | **1.52**  **(1.36, 1.69)** | 0.91  (0.82, 1.02) | **1.26**  **(1.13, 1.41)** |
| Female * Perceived Obesity | **0.81**  **(0.72, 0.92)** | **0.70**  **(0.62, 0.79)** | **0.46**  **(0.41, 0.51)** | **1.65**  **(1.51, 1.86)** | **0.56**  **(0.50, 0.62)** |
| Perceived Underweight | 1.11  (0.91, 1.37) | **1.36**  **(1.16, 1.59)** | **1.32**  **(1.15, 1.52)** | **0.57**  **(0.49, 0.66)** | **1.17**  **(1.01, 1.34)** |
| Female * Perceived Underweight | 1.06  (0.75, 1.47) | 0.78  (0.61, 1.00) | **0.79**  **(0.63, 0.99)** | **2.22**  **(1.79, 2.76)** | **0.72**  **(0.56, 0.94)** |
| DWCB | 0.94  (0.74, 1.14) | **0.77**  **(0.61, 0.98)** | 0.91  (0.73, 1.10) | 0.84  (0.66, 1.04) | **1.14**  **(0.94, 1.39)** |
| Female * DWCB | **0.72**  **(0.55, 0.95)** | 1.16  (0.89, 1.55) | 0.82  (0.63, 1.06) | 1.08  (0.84, 1.40) | **0.62**  **(0.47, 0.80)** |

a) Abbreviations: BMI (body-mass index), MetS (metabolic syndrome), CMR (cardiometabolic risks), WC (waist circumference), FPG (fasting plasma glucose), BP (blood pressure), HDLC (high-density lipoprotein cholesterol), TG (high triglycerides), DWCB (disordered weight control behavior).

b) All models were based on four-level multilevel random intercepts logistic models, in which individuals at level 1 were nested within households at level 2, nested within neighborhoods at level 3, and nested within geographic areas at level 4, adjusting for age (year), BMI (kg/m2), marital status, education, household income, urbanicity, survey year, depression, severe chronic condition (cancer/stroke/coronary heart disease), smoking, drinking, and exercise.

c) Boldface indicates statistical significance (p<0.05)

**Table S2**. Analysis of Interaction Between Perceived Weight and Actual Weight Status Based on Multilevel Logistic Models Predicting Metabolic Syndrome and Cardiometabolic Risk Among Korean Women (N=)

|  | OR  (95% CI) | | | | |
| --- | --- | --- | --- | --- | --- |
|  | MetS | high FPG | high BP | low HDL-C | high TG |
| Perceived Obesity | **1.18**  **(1.04, 1.37)** | 1.03  (0.89, 1.19) | 0.91  (0.79, 1.04) | 1.14  (0.99, 1.30) | 0.99  (0.85, 1.14) |
| Objective Obesity * Perceived Obesity | 1.07  (0.91, 1.28) | 1.08  (0.92, 1.27) | 1.18  (0.98, 1.40) | 0.96  (0.82, 1.14) | 1.05  (0.88, 1.25) |
| Perceived Underweight | 1.04  (0.77, 1.40) | **1.35**  **(1.09, 1.68)** | 1.06  (0.85, 1.30) | 1.08  (0.89, 1.34) | 0.94  (0.73, 1.21) |
| Objective Obesity * Perceived Underweight | 0.95  (0.33, 2.96) | 1.12  (0.40, 2.85) | 0.47  (0.19, 1.20) | 1.16  (0.39, 3.75) | 0.52  (0.16, 1.51) |
| DWCB | **0.59**  **(0.44, 0.80)** | 0.96  (0.77, 1.20) | 1.00  (0.78, 1.26) | **0.77**  **(0.63, 0.94)** | **0.67**  **(0.52, 0.86)** |
| Objective Obesity * DWCB | 1.26  (0.88, 1.87) | 0.96  (0.71, 1.31) | 0.74  (0.53, 1.04) | 1.19  (0.86, 1.60) | **1.44**  **(1.03, 2.02)** |

a) Abbreviations: BMI (body-mass index), MetS (metabolic syndrome), CMR (cardiometabolic risks), WC (waist circumference), FPG (fasting plasma glucose), BP (blood pressure), HDLC (high-density lipoprotein cholesterol), TG (high triglycerides), DWCB (disordered weight control behavior).

b) All models were based on four-level multilevel random intercepts logistic models, in which individuals at level 1 were nested within households at level 2, nested within neighborhoods at level 3, and nested within geographic areas at level 4, adjusting for age (year), BMI (kg/m2), menopause, marital status, education, household income, urbanicity, survey year, depression, severe chronic condition (cancer/stroke/coronary heart disease), smoking, drinking, and exercise.

c) Boldface indicates statistical significance (p<0.05)

**Table S3**. Analysis of Interaction Between Perceived Weight and Actual Weight Status Based on Multilevel Logistic Models Predicting Metabolic Syndrome and Cardiometabolic Risk Among Korean Men (N=9,448)

|  | OR  (95% CI) | | | | |
| --- | --- | --- | --- | --- | --- |
|  | MetS | high FPG | high BP | low HDL-C | high TG |
| Perceived Obesity | **1.24**  **(1.01, 1.49)** | 1.09  (0.90, 1.34) | 1.07  (0.91, 1.25) | 1.16  (0.94, 1.42) | 1.16  (1.00, 1.37) |
| Objective Obesity * Perceived Obesity | 1.23  (1.00, 1.53) | 0.96  (0.78, 1.19) | **1.35**  **(1.13, 1.61)** | 0.90  (0.72, 1.11) | 0.88  (0.74, 1.03) |
| Perceived Underweight | 1.13  (0.90, 1.42) | 0.93  (0.76, 1.14) | 0.98  (0.81, 1.17) | 0.96  (0.77, 1.20) | 0.89  (0.74, 1.05) |
| Objective Obesity * Perceived Underweight | 0.69  (0.27, 1.77) | 1.08  (0.35, 3.22) | 0.90  (0.34, 2.38) | 1.61  (0.57, 4.45) | 1.58  (0.64, 4.07) |
| DWCB | 0.77  (0.45, 1.23) | 0.68  (0.43, 1.11) | 1.24  (0.87, 1.73) | 1.17  (0.81, 1.73) | 0.89  (0.65, 1.23) |
| Objective Obesity * DWCB | 1.28  (0.75, 2.27) | 1.10  (0.60, 1.92) | 0.58  (0.39, 0.89) | 0.68  (0.41, 1.08) | 1.32  (0.88, 1.93) |

a) Abbreviations: BMI (body-mass index), MetS (metabolic syndrome), CMR (cardiometabolic risks), WC (waist circumference), FPG (fasting plasma glucose), BP (blood pressure), HDLC (high-density lipoprotein cholesterol), TG (high triglycerides), DWCB (disordered weight control behavior).

b) All models were based on four-level multilevel random intercepts logistic models, in which individuals at level 1 were nested within households at level 2, nested within neighborhoods at level 3, and nested within geographic areas at level 4, adjusting for age (year), BMI (kg/m2), marital status, education, household income, urbanicity, survey year, depression, severe chronic condition (cancer/stroke/coronary heart disease), smoking, drinking, and exercise.

c) Boldface indicates statistical significance (p<0.05)
